# Effects of introducing isotropic artificial defects on the superconducting properties of differently doped Ba-122 based single crystals

**DOI:** 10.1038/srep27783

**Published:** 2016-06-15

**Authors:** V. Mishev, M. Nakajima, H. Eisaki, M. Eisterer

**Affiliations:** 1 Atominstitut, TU Wien, Stadionallee 2, 1020 Vienna, Austria; 2 Electronics and Photonics Research Institute, National Institute of Advanced Industrial Science and Technology, Tsukuba, Ibaraki 305-8568, Japan

## Abstract

The effects of isotropic artifical defects, introduced via fast neutron (*E* > 0.1 MeV) irradiation, on the physical properties of differently (Co, P and K) doped BaFe_2_As_2_ superconducting single crystals were studied. The Co- and P-doped single crystals showed a second peak in the magnetization curve (fishtail effect) in the pristine state. Significant variations in the radiation-induced changes in the critical current density *J*_c_ were observed in the different types of crystal, while the irreversibility fields did not change remarkably. The highest *J*_c_s were obtained for the K-doped crystal, exceeding 3 × 10^10^ Am^−2^ (*T* = 5 K, *B* = 4 T) and remaining above 8.5 × 10^9^ Am^−2^ at 30 K and 1 T. The pinning force was analyzed to compare the pinning mechanisms of the individual samples. While distinct differences were found before the irradiation, the same pinning behavior prevails afterwards. The pinning efficiency *η* = *J*_c_/*J*_d_ was estimated from the depairing current density *J*_d_. *η* was similar in all irradiated crystals and comparable to the value in neutron irradiated cuprates, suggesting that the huge critical current densities measured in the irradiated K-doped crystal are due to its large depairing current density, making this compound the most promising for applications.

The BaFe_2_As_2_ (Ba-122) based superconductors have interesting properties for applications such as a low upper critical field anisotropy^1^,^2^ and transition temperatures up to almost 40 K. Different doping^3^,^4^,^5^,^6^ results in distinctively different basic properties such as transition temperature *T*_c_, upper critical field and critical current densities. One of the most important properties of a superconductor, in particular when considering its technological relevance, is its critical current density *J*_c_. So far, the highest critical current densities in a Ba-122 based superconductor have been obtained in a P-doped Ba-122 thin film^7^. At present, however, the best wires or tapes based on pnictide superconductors use K-doped Ba-122^8^,^9^,^10^,^11^. Irradiation techniques are an established tool for introducing artificial defects to study their influence on the superconducting properties^12^ and flux pinning. The introduced defects enhance scattering of the charge carriers^13^ which is potentially pair breaking in multi-band superconductors or superconductors having an anisotropic energy gap (e.g. d-wave symmetry in the cuprates). The resulting decrease of *T*_c_^14^ may thus be used to test the pairing symmetry in the iron-based superconductors^15^. Flux pinning, on the other hand, is often largely enhanced after irradiation with the introduced defects being an ideal model system to study pinning and vortex physics or to benchmark the achievable *J*_c_^16^,^17^,^18^,^19^,^20^,^21^,^22^,^23^. In particular, high currents were obtained in Ba-122 single crystals after irradiation with protons and heavy ions^17^,^21^,^22^. A recent report by Taen *et al*.^22^ revealed *J*_c_s above 10^11^ Am^−2^ at 2 K for proton irradiated K-doped Ba-122 single crystals. In a previous study^24^, we have demonstrated the influence of the upper critical field anisotropy on vortex pinning by defects resulting from fast neutron irradiation, while we are focusing here on the differences of pinning in neutron irradiated Ba-122 single crystals arising from the different dopants, which influence basic superconducting properties such as transition temperature, condensation energy, and upper critical field. The depairing current density *J*_d_ varies significantly in the pristine crystals. This results from different intrinsic superconducting properties in the differently doped samples. Neutron irradiation experiments are best suited to distinguish between the two scenarios, since the generated pinning landscape is essentially the same in all the crystals.

## Results

### Transition temperature

*T*_c_ was evaluated from the onset of the diamagnetic response arising from superconductivity (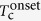). The transition temperatures of all pristine crystals were close to the highest values reported for the respective bulk systems. Thus the crystals can be considered as optimally doped. All samples revealed narrow transition widths, ranging from 0.2 K to 0.7 K as determined from a *T*^10%^ − *T*^90%^ = Δ*T*_c_ criterion, where *T*^10%^ and *T*^90%^ are the respective temperatures at 10% and 90% of the maximum response signal. The difference 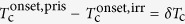 is used to illustrate the effect of fast neutron irradiation on the transition temperature of the differently doped single crystals. The results are summarized in Table 1.

The values obtained in this study agree with previous results on the effects of fast neutron irradiation on *T*_c_ of Co-doped single crystals^25^ and other iron-based compounds^14^. The P- and K-doped crystals show slightly higher decreases in *T*_c_ after irradiation, however, the relative changes are comparable, between 1.6% (K) and 2.4% (P) at 3.6 × 10^21^ m^−2^. Although the absolute decrease of *T*_c_ at this fluence is larger in the cuprates, about 0.9 K, the relative change only amounts to about 1%^26^. In Nb_3_Sn both the absolute (~0.1 K) and the relative (~0.7%) changes are smaller^27^. However, the sensitivity of a superconductor to non-magnetic impurity scattering has to be discussed in terms of scattering rates (or energies), not fluence, since the number and the size of the created defects as well as the scattering rates will not be the same in all the compounds.

### Critical current density

**Figure 1:**
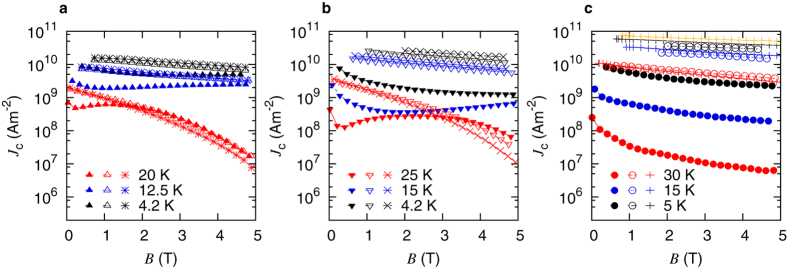
In-plane critical current densities of differently doped Ba-122 single crystals (solid symbols pristine, open symbols Φ_*t*_ = 1.8 × 10^21^ m^−2^, crosshairs Φ_*t*_ = 3.6 × 10^21^ m^−2^); (**a**) Co-doped, (**b**) P-doped, (**c**) K-doped (yellow crosshairs indicate *J*_c_ at 2 K).

The in-plane critical current density was determined in the pristine state and after each irradiation step. The results are shown in Fig. 1. The Co-doped crystal has the highest critical current densities in the pristine state, while the K-doped crystal shows the lowest *J*_c_s prior to fast neutron irradiation. Both the Co- and P-doped crystals exhibit distinctive second peaks in *J*_c_ (fishtail effect) in the pristine state, commonly interpreted as an order-disorder transition of the flux line lattice. Fast neutron irradiation induces different enhancements of *J*_c_ in the three crystals. The changes are much more pronounced after the first irradiation step (to a fluence of 1.8 × 10^21^ m^−2^), while the second irradiation step results in comparatively small changes. Thus, the currents are close to the highest achievable performance for this kind of defects. The Co-doped crystal shows the least pronounced changes in magnitude, while *J*_c_ in the K-doped crystal rises dramatically, by up to a factor of almost 1000 at 30 K and 5 T. The behavior of the critical current density in the P-doped crystal is similar to that observed for the Co-doped crystal. Both of them no longer exhibit a second peak in *J*_c_, since the flux line lattice is now disordered at all fields. However, the P-doped crystal clearly shows larger enhancements of the critical current density after irradiation. The highest *J*_c_ was measured in the the K-doped crystal and reaches values of above 7 × 10^10^ Am^−2^ at 2 K and a self-field of about 0.8 T. The *J*_c_s obtained for the K-doped crystal after irradiation to Φ_*t*_ = 3.6 × 10^21^ m^−2^ are comparable to the previously highest reported *J*_c_s in a Ba-122 based single crystal^17^.

### Irreversibility lines

**Figure 2:**
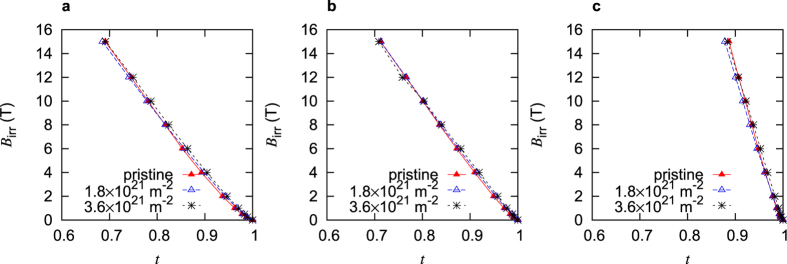
Irreversibility lines (*H*_a_||*c*) of differently doped Ba-122 single crystals (solid symbols pristine, open symbols Φ_*t*_ = 1.8 × 10^21^ m^−2^, crosshairs Φ_*t*_ = 3.6 × 10^21^ m^−2^) as a function of the normalized temperature 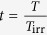; (**a**) Co-doped, (**b**) P-doped, (**c**) K-doped.

The irreversibility lines of the individual crystals were obtained from resistive measurements (see Section 4 for more details). Figure 2 shows the influence of fast neutron irradiation. The irreversibility field *B*_irr_ is plotted versus the normalized temperature *t* = *T*/*T*_irr_, where *T*_irr_ is determined from resistive measurements at zero applied field by the 1 *μ*V/cm criterion. Note that in all these cases the pristine crystals are not the same as the crystals which were irradiated with fast neutrons, but they originate from the same synthesis batch. Within the resulting uncertainties, *B*_irr_(*t*) of all crystals does not undergo remarkable changes after irradiation. Note that the pristine K-doped crystal exhibits by far the largest irreversibility fields while the critical current density at high temperatures (*t* > 0.8) is much lower than in the other two crystals in the pristine state as was already shown. In addition, it was found that the upper critical fields also did not show significant changes after irradiation.

## Discussion

The magnitudes of the critical current densities obtained in the irradiated crystals can be traced back to the depairing current density, *J*_d_, in the respective system. It is given by the following equation:


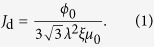


Here, *λ* and *ξ* refer to the penetration depth and coherence length within the *ab*-planes. The values at 0 K were obtained from literature. To establish a correlation between *J*_d_ and *J*_c_, the pinning efficiency parameter *η* = *J*_c_(4.2 K, *H*_a_ = 0)/*J*_d_ is introduced. The results for all crystal types are shown in Table 2 after irradiation to Φ_*t*_ = 3.6 × 10^21^ m^−2^.

**Figure 3:**
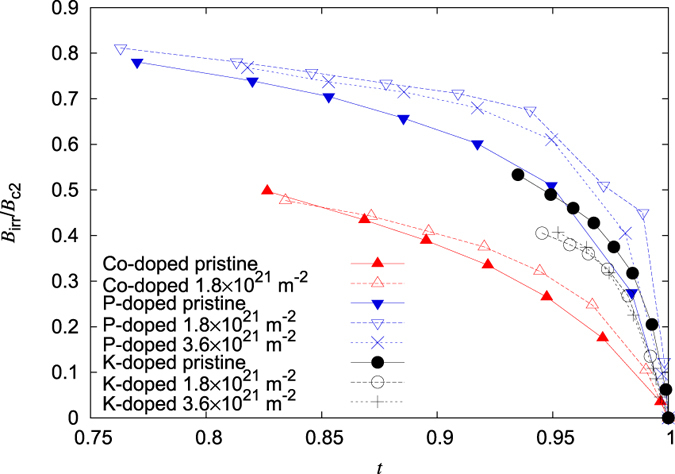
Normalized irreversibility field 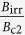 as a function of 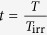 in the differently doped single crystals before and after fast neutron irradiation.

The pinning efficiency is between about 2% and 5% for the differently doped crystals, whereby the K-doped crystal has *η* = 3.5%. Note that *J*_c_ at self field is used for the evaluation of *η*, which is around 0.7 T for the K- and Co-doped crystals and around 2 T for the P-doped crystal. The sharp peak in the doping dependence of *λ*, reported by Hashimoto *et al*.^28^ at optimal doping is the reason for the distinctively lower *J*_d_ in the P-doped crystals and may be related to the higher *η*. Either the large *λ* fosters a high *η* or our crystal does not have the exact stochiometry at which the peak occurs and the large calculated value of *η* results from an overestimation of *λ*. An interesting correlation to these values of *η* in the three differently doped crystals can be found in Fig. 3, which shows the irreversibility fields normalized by the values for *B*_c2_ and plotted versus the normalized temperature 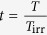. The results for the irradiated crystals are of particular interest since they are expected to have nearly identical, isotropic defect structures after the fast neutron irradiation. Figure 3 shows that the P-doped crystal has the highest value of *B*_irr_/*B*_c2_ after irradiation, while the Co-doped crystal has the lowest. The correlation with the obtained *η*s supports the notion that the pinning efficiency is highest in the P-doped crystal and lowest in the Co-doped crystal. An interesting explanation for the efficient flux pinning in the P-doped system is based on quantum fluctuations, which may enhance the core energy of vortices^29^ and thus increase the pinning force^30^.

Similar *η*s of 2–3% can be derived for neutron irradiated cuprate based superconductors^31^,^32^. They are reasonable when considering the fact that fast neutron irradiation creates spherical defects with radii ranging from several nanometers to point-like defects. A defect structure consisting of linear continuous cylindrical defects with radii similar to the coherence length of the superconductor can lead to much higher *η*s of around 0.2^33^, which establishes an upper limit for critical currents caused by de-pinning. The presently highest critical currents measured in a Fe-based superconductor^16^ were obtained after columnar defects had been created by heavy ion irradiation. The report by Fang *et al*. presents data on an irradiated Sm-1111 crystal with *J*_c_(5 K) ≈ 2 × 10^11^ Am^−2^. *J*_d_ ≈ 16.8 × 10^11^ Am^−2^ is calculated from *λ* = 200 nm and *ξ* = 1.5 nm^34^,^35^. The resulting *η* is about 12%, therefore distinctively higher than the *η* obtained for the fast neutron irradiated K-doped Ba-122 crystal. It should also be noted that the depairing current densities for the 1111 crystal and the K-doped Ba-122 are very similar. This means that critical current densities close to those reported for the 1111 crystal by Fang *et al*. should be attainable in K-doped Ba-122 single crystals if an optimized defect structure is created. Previous heavy ion irradiation experiments on K-doped Ba-122 single crystals^17^,^21^ did not result in critical current densities similar to those in the 1111 system but they are comparable to those in the present work. This might be related to the quality of the crystals used in those studies, where *T*_c_ was almost 1 K lower in the pristine state, possibly resulting in a smaller *J*_d_.

**Figure 4:**
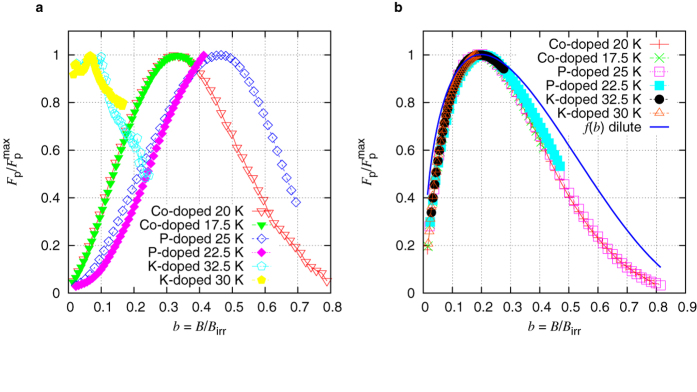
Normalized volume pinning force as a function of the reduced field in the three differently doped Ba-122 single crystals; (**a**) Pristine, (**b**) Irradiated.

A nearly identical pinning mechanism in all irradiated crystals, despite the obvious differences in the magnitude of the critical current densities of the individual crystals, is illustrated by the volume pinning force *F*_p_ = *J*_c_*B*. Figure 4 shows the normalized bulk pinning force 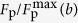 where 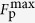 is the maximum pinning force measured on the respective crystal at a certain temperature and *b* = *B*/*B*_irr_ is the reduced field. It should be noted that the results shown in the plot refer to similar normalized temperatures *T*/*T*_c_. The differences in the pristine crystals (Fig. 4a)) are very pronounced and further illustrate the dependence of the pinning mechanism on the dopant.

The introduction of a nearly identical isotropic defect structure in all three crystal types results in significant changes in the behavior of *F*_p_. Figure 4b illustrates how the curves collapse onto a single curve. This result confirms the assumption that the introduced strong pinning centers are the dominant factor in determining the pinning properties of the individual crystals and renders the pronounced differences in the pristine state marginal. To compare these curves with theory, the expected behavior of *F*_p_ in the limit of dilute pinning is plotted in Fig. 4b (solid blue line)^36^. It is given by the following equation:


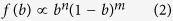


where n = 0.5 and m = 2. While the results do not follow the behavior predicted by this limit at all *b*, it is remarkable that the position of the peak in 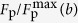 matches *b* = 0.2 as predicted by *f *(*b*). This means that the reduced induction *b*, where *F*_p_ reaches its maximum, is correctly predicted by this model, which is valid for pinning by strong but dilute pins.

In conclusion, the introduction of an isotropic artificial defect structure in all crystals resulted in basically identical normalized pinning force curves for all three doping types. The defect structure resulting from fast neutron irradiation thus dominates flux pinning. The position of the peak in the pinning force is predicted correctly by the dilute pinning limit. The highest critical currents were obtained in an irradiated K-doped Ba-122 single crystal because of its high depairing current density. This reveals the high *J*_c_ potential of this compound. The irradiated P-doped crystal on the other hand, shows the highest pinning efficiency, which may be related to the sharp maximum in *λ* at a P-concentration of *x* = 0.3 (measured for this system by Hashimoto *et al*.^28^), and the occurence of quantum fluctuations. As regards possible technological applications combined with the fact that the best superconducting wires based on pnictides use K-doped Ba-122, the results of this paper provide a stimulus for further optimization of the pinning structure in this material with the goal of reaching higher critical current densities.

## Methods

The single crystals were grown at the National Institute for Advanced Industrial Science and Technology in Tsukuba, Japan. They were synthesized by the self-flux method at optimal doping level^37^,^38^,^39^. Their nominal chemical compositions are Ba(Fe_0.94_Co_0.06_)_2_As_2_, BaFe_2_(As_0.7_P_0.3_)_2_ and Ba_0.6_K_0.4_Fe_2_As_2_. All crystals were of cuboidal shape with typical dimensions of about 2 × 2 × 0.1 mm^3^, which were determined by an optical microscope and from the mass and the theoretical density. The *T*_c_s were determined from the onset of diamagnetic shielding in ac susceptibility measurements using a 1 T SQUID (field amplitude of 0.1 mT and orientation parallel to the crystal’s *c*-axis). The in-plane critical current densities were established from magnetization measurements at different temperatures in a 5 T vector VSM and a 7 T SQUID, with the applied field *H*_a_ aligned parallel to the *c*-axis. The evaluation procedure is based on the isotropic Bean model^40^ and takes the self-field of the sample into account^41^. The critical current density is calculated from the irreversible magnetic moment *m*_irr_ (obtained from magnetization loops) and the geometry of the individual crystals. The upper critical field 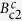 and the irreversibility lines 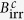 (*H*_a_||*c*-axis) of the individual crystals were established from resistive measurements by applying a current density of about 3 × 10^4^ Am^−2^ in applied magnetic fields of up to 15 T. *B*_c2_ was defined by a 10% drop in resistivity (i.e. near the onset of the transition), *B*_irr_ by an electric field criterion of 1 *μ*Vcm^−1^, which was very close to the “zero” resistivity field (offset of transition). A standard four-probe configuration (with indium press contacts) was used for these measurements. The fast neutron irradiation was carried out in the TRIGA-MARK II reactor in Vienna. The resulting fast neutron (*E* > 0.1 MeV) fluence Φ_*t*_ was determined via activation analysis using nickel monitors which were placed in the same quartz tubes as the crystals.

## Additional Information

**How to cite this article**: Mishev, V. *et al*. Effects of introducing isotropic artificial defects on the superconducting properties of differently doped Ba-122 based single crystals. *Sci. Rep.* **6**, 27783; doi: 10.1038/srep27783 (2016).

## Figures and Tables

**Table 1:** *T*_c_s and transition widths of the single crystals before and after irradiation.

Sample	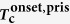 (K)	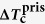 (K)	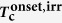 (K)	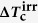 (K)	*δT*_c_(K)	Φ_*t*_(10^21^ m^−2^)
Co-doped	24.2	0.7	24.05	0.7	0.15	1.8
— ” —	— ” —	— ” —	23.8	0.79	0.4	3.6
P-doped	29.4	0.4	29.1	0.5	0.3	1.8
— ” —	— ” —	— ” —	28.7	0.89	0.7	3.6
K-doped #1	38.25	0.1	–	–	–	–
K-doped #2	38.4	–	38.2	0.2	0.2	1.8
— ” —	— ” —	— ” —	37.8	0.39	0.6	3.6

**Table 2:** Depairing current density and pinning efficiency after fast neutron irradiation to a fluence of 3.6 × 10^21^ m^−2^.

Sample	*ξ* (nm)	*λ* (nm)	*J*_d_ (10^11^ Am^−2^)	*η* (%)
Ba(Fe_0.94_Co_0.06_)_2_As_2_	2.7^42^,^43^	200^44^	9.3	1.7
BaFe_2_(As_0.7_P_0.3_)_2_	2.14^45^	300^28^	5.2	5
Ba_0.6_K_0.4_Fe_2_As_2_	1.88^46^	180^47^	16.6	3.5
